# Supplementary material for: Suicide capability within the ideation-to-action framework: A systematic scoping review
Source: PLoS One. 2022 Oct 27;17(10):e0276070. doi: 10.1371/journal.pone.0276070 (PMC9612581; doi:10.1371/journal.pone.0276070)
Supplement: S1 Table — This table provides the aim of each article and how findings are linked to the concept of suicide capability, where applicable. (DOCX) [file pone.0276070.s001.docx]

**S1 Table. Study aims and theoretical relationships.** This table provides the aim of each article and how findings are linked to the concept of suicide capability, where applicable.

| Reference | Research aim (verbatim where possible) | Findings regarding suicide capability linked to theory |
| --- | --- | --- |
| Abdollahpour Ranjbar et al. [135] | “The primary objective of the present study was therefore to investigate whether components of cognitive control and cognitive emotion regulation differ among Iranian women with MDD with either prior suicide attempts or suicidal ideation only (i.e., without prior suicide attempts) and healthy controls”. | Deficits in cognitive control may contribute to cognitive distortions which are associated with suicidality. |
| Allbaugh et al. [85] | “This study examines the direct links between three types of childhood abuse (physical, sexual, emotional) and suicide resilience and the mediational roles of the three IPTS components in these links”. | Greater severity of childhood physical abuse predicted higher levels of acquired capability for suicide. |
| Ammerman et al. [119] | The current study examined three characteristics of NSSI (frequency, number of methods, and subjective pain) that may help to elucidate the relationship between NSSI and acquired capability. | The absence of pain during NSSI may indicate acquired capability. |
| Anestis & Joiner [111] | “To test the role of negative urgency in the interpersonal–psychological theory”. | While experiencing negative affect the propensity to engage in suicidal behaviour when all three theory components are elevated is substantially higher. |
| Anestis et al. [112] | “To further clarify the specific nature of the contribution of negative urgency to suicidal behaviour”. | Clarifies the role of negative urgency and links to the acquired contributor. |
| Anestis et al. [58] | “We hypothesized that SUD patients with BPD would report more frequent suicide attempts (overall and across varying levels of suicidal intent and medical severity) than those without BPD. Furthermore, we expected that the association between BPD and suicide attempts would be moderated by DT, such that BPD patients with high DT would report a greater frequency of suicide attempts (including medically-severe suicide attempts) than those with low DT”. | The extent to which psychological distress tolerance manifests as the physical pain component of acquired capability remains unclear. |
| Anestis et al. [106] | “We hypothesized that the 3-way interaction between perceived burdensomeness, thwarted belongingness, and the acquired capability for suicide would predict lifetime suicide attempts”. | Results indicate that acquired capability contributes to the movement from suicidal desire to suicide attempts. |
| Anestis et al. [133] | “To examine whether such legislation (universal background checks and mandatory waiting periods) is associated with sustained changes in the trajectory of statewide suicide rates across time”. | Not linked |
| Aschrafi et al. [142] | “Postmortem brain samples of the rostro-ventral midbrain of depressed suicide completers were examined for altered miRNA levels in stress response.  Using this approach, we tested the hypothesis that miRNAs would directly modulate stress-induced neuropeptide expression, resulting in the identification of selective miRNAs as upstream modulators of these neuropeptides. This postulate was tested in a stress-sensitive model system, the midbrain urocortin 1 (Ucn1) neuronal system”. | Not linked |
| Baer et al. [99] | “To examine the role of ITS variables in the association between substance use frequency in general and suicidal ideation and suicide attempts; to determine the unique relevance of ITS variables in the association between substance use frequency and suicide out- comes above and beyond more general negative self-directed cognitions, analyses were repeated with self-disgust”. | Advances understanding of potential links between substance use and acquired capability. |
| Baertschi et al. [104] | “The aim of this study was to explore the IPTS in the context of suicide-related thoughts and behavior of varying lethality, ranging from passive suicidal ideation to attempted suicide”. Hypothesis 3 is formulated as follows: “the simultaneous presence of suicidal desire and lowered fear of death serves as the condition under which suicidal desire will transform into suicidal intent”. | Fearlessness about death did not differentiate suicide attempters from suicide ideators. |
| Ben‐Efraim et al. [121] | “We investigated physical attack (PA), rape or cumulative lifetime stressful life events (SLE) exposures in gene-environment interactions (G×E) with CRHR1 polymorphisms in a large family-based sample with SA offspring and in a case-control comparison with healthy volunteers”. | Not linked |
| Beyond Blue [44] | “The purpose of the study was to identify contributors and warning signs for suicidality among men, and factors that may prevent or interrupt suicidal behaviour”. | Not linked |
| Biddle et al. [45] | “This study explored the factors influencing the decision to use or contemplate hanging as a method of suicide, with the aim of identifying approaches to prevention that may be developed to reduce its popularity”. | Not linked |
| Blankenship [63] | “What are the relationships between trauma type (i.e., interpersonal assaultive, interpersonal non-assaultive, and noninterpersonal), age at time of trauma exposure, level of PTSD symptomology, and suicide-related outcomes (i.e., SI, SA, and SSRO)?  What are the relationships between trauma type, age at time of trauma exposure, PTSD symptomology, IPTS constructs of PB, TB, and AC (Joiner, 2005), and suicide-related outcomes (i.e., SI, SA, and SSRO), and how do PB, TB, and AC interact with trauma type?” | Acquired capability was not significantly correlated with interpersonal trauma; acquired capability was not a significant predictor of suicide attempts after including interaction effects from trauma types, however perceived burdensomeness was. Prior theory does not explain these relationships. |
| Brackman et al. [120] | “The primary purpose of this study was to investigate how the association between suicidal ideation and attempts would be differentially moderated by NSSI frequency as compared to the acquired capability variables of pain tolerance and fearlessness about death in a college sample. We hypothesized that NSSI frequency and combined fearlessness of death/pain tolerance would separately moderate the association between suicide ideation and attempts. In addition, we were interested in exploring the association between NSSI and fearlessness about death given the paucity of information on that relationship”. | Results suggest that NSSI frequency can increase suicide risk distinct from pain tolerance or fearlessness about death. |
| Calati et al. [76] | An association study was conducted on the catechol-O-methyltransferase gene (COMT) and potential links between seven COMT polymorphisms (rs737865, rs5844402, rs5993883, rs4680, rs4633, rs165599 and rs9332377) on both personality traits and suicidal behavior. | Not linked |
| Cao et al. [84] | “To investigate capability of daily living activities and family function preceding death among elderly suicide cases and to explore associations between disability in daily living activities, family dysfunction, and late-life suicide in rural China.” | Not linked |
| Carli et al. [130] | “To explore the role of impulsivity in prisoners' suicide behaviour”. | Not linked |
| Cheek et al. [72] | “The current study aimed to address this limitation in providing a stringent empirical test of whether a risk factor for the acquired capability for suicide is specific to suicide attempts and not SI in a nationally representative sample of adults with major depression. Specifically, we examined injections drug use in relation to SI, plans, and attempts. Drawing on the interpersonal theory of suicide, we hypothesized that injection drug use would be positively associated with suicide attempts, but not SI or plans. Further, we hypothesized that among ideators, injection drug use would be associated with elevated risk for suicide attempts but not plans. Lastly, within the subsample of individuals who have made a suicide plan, injection drug use was hypothesized to be associated with increased odds of suicide attempts”. | Intravenous substance use may be considered an example of a painful event that could contribute to acquired capability. |
| Chelmardi et al. [67] | “To explore the prediction of suicide ideation formation and its transition to suicide attempt through the interactions of the constructs suggested by the IPTS, IMV and 3ST theories, as well as other clinical factors”. | Provides evidence for some of the core constructs from the theories. |
| Chu et al. [62] | “This study examined whether major depressive episodes (MDEs) may be particularly relevant to suicidal behavior when considered in the context of AC”. | Acquired capability may distinguish depressed military personnel suicide ideators from depressed military personnel suicide attempters. |
| Chu et al. [77] | “This study investigated thwarted belongingness (TB), perceived burdensomeness (PB), and capability for suicide (CS) as explanatory links in the association between NSSI, ideation, and suicide attempt history”. | NSSI appears to contribute to acquired capability. |
| Copeland et al. [89] | “The aim of this study is to test whether childhood STBs are related to adult psychiatric, suicidal, and functional outcomes”. | Not linked |
| Daruwala et al. [105] | “The current study will seek to clarify the relationship between masculine traits and the components of the ITS”. | Sensation seeking and stoicism contributed to acquired capability fearlessness about death, but stoicism also contributed to thwarted belongingness. |
| DeVille et al. [126] | “To test the hypothesis that abnormalities of interoception are associated with suicidal capacity in individuals with psychiatric disorders”. | Aligns with ideation-to-action theoretical models by differentiating controls from suicide attempters. |
| Dhingra et al. [68] | “To empirically test the Three-Step Theory (3ST) in a sample of university students”. | Supports the relevance of dispositional and practical contributors in addition to acquired capability. |
| Duddin & Raynes [46] | “To utilize the unique access to suicide notes written by those who died by suicide on the railway so as to gain a greater understanding of why people chose this method”. | Not linked |
| Feltrin et al. [93] | “The present study aims at analyzing the impact of stress on quality of life and health behaviors of multiprofessional residents of a university hospital”. | Not linked |
| Forrest et al. [56] | “We investigated interoceptive abilities in individuals with differing degrees of suicidality”. | Interoceptive deficits and acquired capability may combine to create an increased risk for suicide attempts. |
| Govind (Study 2) [47] | “To examine whether any specific psychological factors influence the choice for a violent method of suicide such as self-immolation”. | Results suggest that traumatic experiences, previous suicide attempts and self-harm contribute to acquired capability. |
| Hardt et al. [82] | “To systematically compare reports from subjects who grew up in Poland with those who grew up in Germany during this period. In addition, childhood sexual and physical abuse as risk factors for suicide attempts and alcohol abuse were compared in both countries”. | Not linked |
| Heiden‐Rootes et al. [97] | To explore “the impact of religious and nonreligious gender identity change efforts (GICE) on the mental health of transgender and nonbinary adults”. | Not linked |
| Hsiao et al. [139] | To present a case report a 63-year-old male patient with semantic dementia who was hospitalised after multiple suicide attempts. | Not linked |
| Huang et al. [117] | “We likewise hypothesize that the differences between people who engage in NSSI and people who attempt suicide are complex rather than complicated or simple. We accordingly hypothesize that no simple or complicated algorithm will be necessary and sufficient to correctly distinguish between all (or nearly all) people who engage in NSSI and suicide attempts.  The present study will test this hypothesis by evaluating whether any simple or complicated algorithms are necessary and sufficient to distinguish between people who engage in NSSI and suicide attempts.”. | Acquired capability for suicide yielded chance level accuracy; results suggest that it is unlikely for an individual factor or a small set of individual factors to be both necessary and sufficient to distinguish between individuals engaging in NSSI and suicide attempt. |
| Joiner et al. [115] | “To test the three-way interaction of the IPTS constructs”. | Not linked |
| Jordan et al. [98] | “The purpose of the present study was to fill this gap in the literature by testing the hypotheses that (1) PPE are associated with suicide intent and (2) that PPE mediate the relationship between impulsivity and suicide intent”. | Three-way interaction of perceived burdensomeness, thwarted belongingness, and acquired capability differentiated suicide attempters from suicide ideators. |
| Jordan & Samuelson [101] | “In this study we aimed to address these limitations by 1) comparing individuals who made a suicide attempt with high intent to those who made a cry for help, and (2) examining a broader range of trauma, such as committing violent acts.  We hypothesized that repeated exposure to violent trauma would predict suicide intent, more so than other forms of trauma”. | Painful and provocative events, but not impulsivity, were a significant predictor of intent to die among suicide attempters. |
| Jovičić et al. [127] | “The primary aim of this study was to determine whether affective temperaments and personality (character) traits can predict suicide attempts in population of patients with depression”. | Results indicate that committing violence, but not trauma types as a victim, distinguished between suicide attempters who did or did not want to die. |
| Kasen et al. [128] | “In the current study, we investigated features of impulsivity and capability as related to suicide attempt with longitudinal data drawn from a large community sample of individuals followed in multiple waves from childhood into adulthood”. | Not linked |
| Kene [78] | “The present study examined the role of theoretical constructs – acquired capability for suicide, reasons for attempting suicide (internal perturbation based reasons vs. extrapunitive/manipulative reasons), implicit identification with self-injury, and implicit attitude towards self-injury”. | Acquired capability did not differentiate between suicide ideators and suicide attempters. |
| Kerbrat et al. [86] | “The objective of the present study was to assess the impact of gender, suicide attempts, and combat deployments on self-reported acquired capability for suicide in a large sample of active-duty service members with current or recent suicidal ideation (with or without history of suicide attempt)”. | Results suggest that frequency of deployments to combat zones increases acquired capability. |
| Khazem & Anestis [17] | “The aim of this research is to identify factors distinguishing between those who have and have not acted on suicidal thoughts”. | History of painful and provocative events differentiated between suicide attempters and suicide ideators; fearlessness about death did not. |
| Kishikawa et al. [140] | “To illustrate the influence of sociopsychological background on behaviour in patients with Alzheimer’s disease”. | Not linked. |
| Klonsky et al. [116] | “Our primary aim was to determine the strength of the association between NSSI and attempted suicide on four diverse samples: adolescent psychiatric patients, adolescent high school students, university undergraduates, and a random-digit dialling sample of U.S. adults”: | Results suggest that NSSI may contribute to both acquired capability and suicidal ideation. |
| Knowles et al. [124] | “We aimed to evaluate the extent of the genetic overlap between suicide attempt and cholesterol”. | Not linked |
| Koweszko et al. [144] | “The objective of this study was to explore the role of oxidative stress components in suicidality comparing subjects at different stages of suicide”. | Some stress components may contribute to the behavioural shift from the motivational stage of the IMV to attempting suicide. |
| Kunde et al. [48] | “The current study aims to draw upon the IPT and utilises psychological autopsy (PA) information to create lifecharts in order to explore the pathways to suicide and suicidal process in Australian farmers”. | Painful and provocative events such as euthanising animals appears to contribute to acquired capability. |
| Law & Anestis [103] | “To test the differential effects of rumination in the context of a high arousal (anger) vs. low arousal (sadness) emotional state on changes in suicide capability using two undergraduate student samples”. | Ability to tolerate and persist through pain did not result in changes of suicide capability. |
| Law et al. [132] | “This investigation aims to evaluate the short- and long-term effect of safety barriers on Brisbane’s Gateway Bridge and to examine whether there was substitution of suicide location”. | Not linked |
| Law et al. [118] | “The aim of the present studies is to determine the role of persistence through pain and distress on the relationship between NSSI and past suicide attempts”. | Pain persistence and/or distress tolerance may mediate the relationship between NSSI and suicide attempts within suicide capability. |
| Leira et al. [71] | “To compare characteristics between self-harm and suicide in the same population to reveal differences and similarities”. | Not linked. |
| Li et al. [122] | “The aim of this study was to determine if three biomarkers for suicide attempts previously identified and replicated in a genome-wide association (GWAS) study of bipolar disorder (BD) suicide attempters also predicted suicide attempts in patients prospectively diagnosed with schizophrenia (SCZ) or schizoaffective disorder (SAD)”. | Not linked |
| Liu [74] | “This study investigates examines four critical questions:  (1) what are the differences between Asian youth' suicide ideation/attempt and White youth?  (2) What is the relationship between parental support, exposure to suicide behavior, peer relations and religion and suicide ideation/attempt for Asian youth?  (3) To what degree do other factors such as age, marital status, education, economic status contribute to suicide ideation/attempt among this population? ( 4) What are the different characteristics between Asian adolescents' suicide ideation/attempt and Asian young adults' suicide ideation/attempt?” | Not linked |
| Love & Durtschi[73] | “To describe characteristics of individuals with and without suicidal ideation or behaviors in a nationally representative sample of young adults.  This study addressed two primary research questions:  1) How many profiles of risk and protective factors are the best fit for this representative sample of young adults, and what are the characteristics of these profiles?; and  2) Which profiles are associated with increased or reduced risk for suicidal ideation and behaviors?” | Results suggest that acquired capability via previous suicide attempts and as a victim of caregiver-perpetrated child abuse increases risk for suicide attempts. |
| Martin [52] | “The goal is to present a plausible statement about Hemingway’s complex psychiatric picture”. | Not linked |
| McCarthy et al. [138] | “To examine vulnerabilities for mental illness and selfharming behaviours among male prisoners screening positive for a range of neurodevelopmental difficulties - including but not confined to disorders of intellectual ability, attention deficit hyperactivity, and in the autistic spectrum”. | Not linked |
| Medeiros et al. [134] | “This study aimed to evaluate the coping orientations, executive functioning, attentional capacities and decision-making capabilities of a group of depressed suicidal patients and compare the results with a depressed non-suicidal group”. | Acquired capability most frequent in the narratives. |
| Miller [114] | “To explore how suicide risk factors and the dominant theories of suicide are represented in the narratives of suicide attempt survivors to examine whether some theories of suicide occur more frequently in the narratives than others”. | Not linked. |
| Oakes-Rogers & Slade [92] | “The purpose of this paper is to explore the role of trauma experience in pathways to self-harm or attempted suicide in female prisoners who died through self-inflicted death in England and Wales”. | Physical or violent abuse trauma linked to an increasing number of suicide attempts before suicide. |
| Olié et al. [137] | “We studied clinical and neuropsychological profiles of suicide attempters relative to non-attempters, while taking into account the severity of suicide attempt”. | Not linked |
| Oshnokhah et al. [143] | “To investigate the correlation between oxidant-antioxidant levels and suicidal behavior in Kurdish ethnicity in Ilam province”. | Not linked |
| Pelton et al. [113] | “Examining how the ITS explains suicide in autistic people and comparing this to non-autistic people”. | Acquired capability as fearlessness about death only indirectly significant with suicide attempters through traumatic events. |
| Pettit et al. [88] | “To examine the moderating effects of very early onset diagnostic status (≤ 13 years) upon the association between life events and non-fatal suicide attempt”. | Not linked |
| Pisetsky et al. [81] | “The current study examined the extent to which the IPTS components are associated with lifetime suicidal ideation (SI) and lifetime suicide attempts (SA) in a heterogeneous ED sample”. | Painful and provocative events associated with suicide attempters; fearlessness about death not associated with suicide attempters. |
| Pitman et al. [131] | “We aimed to explore the potential for means restriction interventions in a sub-group of psychiatric patients with co-morbid physical illnesses”. | Not linked. |
| Price [80] | “This research presents an opportunity to re-evaluate the current theories by which we understand suicide, and whether this population of inmates who have completed suicide fits within that existing framework”. | History of lethal attempts greater than participants who died by suicide than participants with a history of a single attempt; habituation to pain, death, and dying greater than for single attempters than for those who died by suicide. |
| Rappaport et al. [75] | “The present study examined the correlates of suicidal ideation within a sample diagnosed with major depressive disorder (MD) and the correlates of attempted suicide within those participants who reported suicidal ideation”. | Not linked. |
| Raubenheimer & Jenkins [49] | “To identify the main factors that cause patients to attempt suicide in the Eden district and then attend the Emergency centre”. | Not linked |
| Richard-Devantoy et al. [136] | “We aimed at assessing alterations in cognitive inhibition, a suspected major mechanism of the suicidal vulnerability, in suicidal depressed elderly”. | Not linked. |
| Rogers et al. [57] | “The aim of the current study was to explore relationships between specific facets of exercise dependence, capability for suicide, and past suicide attempts”. | Exercising despite pain and exhaustion may be contributing to acquired capability as a painful and provocative event. |
| Ryan et al. [83] | “To examine young adults’ retrospective reports of parent-initiated efforts to change their sexual orientation during adolescence, and the associations between these experiences and young adult mental health and adjustment”. | Not linked |
| Shelef et al. [94] | “To shed light on the role of dissociation and habituation as facilitators of suicidal behavior, beyond other well-established risk factors of stress, such as depression and hopelessness”. | Dissociation and acquired capability distinguished suicide attempters from controls. |
| Shelef et al. [87] | “The purpose of this study is to examine the role of two types of stressors in intent to die by suicide in the Israeli Defense Force (IDF)”. | Perceived stress predicted intent to die greater than suicidal ideation and acquired capability. |
| Shim et al. [109] | “To test the applicability of the interpersonal–psychological theory of suicide to community-dwelling older persons in South Korea”. | Acquired capability moderated the relationship between suicidal ideation and suicide attempts. |
| Smith et al. [90] | “The purpose of this study was to examine the fearlessness component of the acquired capability for suicide using self-report assessment instruments and an objective measure of aversion (the affectively modulated startle reflex task)”. | Results suggest that painful and provocative events are more important than common negative life events for the development of acquired capability. |
| Smith et al. [63] | Study 1: “over-exercise is uniquely associated with suicidal behavior in BN participants, over and above other types of compensatory mechanisms”  Study 4: “acquired capability for suicide accounts for the relationship between over-exercise and suicidal behavior” | Over-exercise appears to contribute to acquired capability. |
| Smith et al. [102] | “The objective of the current study was to examine the relations between the rate of physiological habituation and the acquired capability for suicide relative to other risk factors for suicide”. | Results suggest that more frequent painful and provocative events were not associated with habituation and in turn, acquired capability. |
| Sokolowski et al. [79] | “To gain better insight, we here conducted a family-based association study of 113 SNPs located across all NMDAR genes”. | Not linked |
| Stoliker & Abderhalden [100] | “To assess a range of sociodemographic and clinical factors (potentially) linked to suicidal ideation, as well as the transition from ideation to attempt, among individuals who are incarcerated in jail”. | Findings suggest that factors related to suicidal ideation are different from factors linked with suicide attempt. |
| Sunnqvist et al. [51] | “The aim of this study is to explore whether a time-geographic life charting, combined with a survey of a person’s coping capacities over time, elucidates the pathway to suicidal behaviour”. | Not linked |
| Suto & Arnaut [50] | “Our aim was to contribute to the knowledge base about factors associated with suicide in prison”. | Not linked |
| Tull et al. [123] | “The goal of this study was to examine the interactive association of borderline personality disorder symptoms and the COMT Val Met polymorphism with past-month suicidal ideation and lifetime suicide attempts”. | Findings suggest that individuals with the Val/Val variant of the Catechol-o-methyltransferase Val159Met polymorphism were more likely to have a lifetime suicide attempt than other participants. |
| Van Orden et al. [60] | “The current studies were designed to test the theory’s two main domains—the role of thwarted belongingness and perceived burdensomeness in suicidal desire (Study 1) and the acquired capability for suicide (Study 2), as well as the joint influence of suicidal desire and the acquired capability (Study 3)”. | Greater numbers of suicide attempts and painful and provocative events were associated with higher levels of acquired capability. |
| Van Orden et al. [91] | “The current study examined the relations of the major constructs of the interpersonal theory with suicide case status compared to living controls in the second half of life.  The current paper tests the IPTS with regards to the second half of life”. | Painful and provocative events significant predictor of death by suicide compared to controls. |
| Wolford-Clevenger et al. [108] | “The purpose of the present study was to test whether the IPTS constructs associate with suicide ideation and attempts among men and women arrested for domestic violence and mandated to Batterer Intervention Programs (BIPs)”. | Acquired capability did not differentiate between suicide attempters and suicide ideators. |
| Wolford-Clevenger et al. [65] | “The purpose of the current study was to test whether an empirically supported theory of suicide, the IPTS, may explain the increased risk of suicide ideation and attempts in women seeking shelter from IPV”. | Findings suggest that acquired capability may develop from being the perpetrator of violence as opposed to being a victim. |
| Wong et al. [129] | “Investigate the extent to which psychiatric, psychological, socio-economical, and life adversities might have an effect on the decision of taking one's life for the middle-aged group”. | Not linked |
| Yang et al. [69] | “The aim of this study was to test the Three-Step Theory (3ST) of suicide in a sample of college students in China”. | Suicide capability as a combination of the three contributors differentiated suicide attempters from suicide ideators as did practical; dispositional and acquired did not. |
| Zhao et al. [95] | “We used detailed information from a large study of medically serious suicide attempters in China to compare the characteristics of suicide attempters across four age groups”. | Not linked. |
