# Supplementary material for: Suicide capability within the ideation-to-action framework: A systematic scoping review
Source: PLoS One. 2022 Oct 27;17(10):e0276070. doi: 10.1371/journal.pone.0276070 (PMC9612581; doi:10.1371/journal.pone.0276070)
Supplement: Attachment — Submitted filename: Authors reply letter.docx [file pone.0276070.s003.docx]

**Author’s reply to original reviewer comments**

We thank the reviewers for taking the time to review our manuscript and providing comments that helped provide a more up-to-date and comprehensive picture of suicide capability. That is, we have taken on board many of the helpful comments and suggestions and incorporated these into the revised and updated review.

| Original comment | Authors’ reply |
| --- | --- |
| Reviewer 1 |  |
| Despite the laudable intentions of the authors, the manuscript is not without several limitations that makes this review highly question the quality of the work produced, especially given that another group of authors have released a review on suicide capability (Gallyer, A. J., Hajcak, G., & Joiner, T. E. (2020). What is Capability for Suicide? A Review of the Current Evidence.). While independent research groups can produce reviews at the same time, knowledge of another review makes it difficult to not engage in any comparisons of the contents produced, and while the focus slightly does differ between the current manuscript and Gallyer et al.’s review, this reviewer has reached the conclusion that the current manuscript is comparatively lacking in terms of quality compared to Gallyer et al. for several reasons. | Thank you for bringing this review to our attention. We had overlooked this as this is a preprint review located on a preprint server. We have now read this review, corresponded with the lead author, and discussed it in our manuscript. We feel the focus of our scoping review is significantly different to Gallyer et al. including its different methodological approach. With that, our review differs from the Gallyer et al. review in three critical ways.  First, our now 90-article review focuses on suicide capability within the ideation-to-action framework that is comprised of multiple contributors from all three theories within the framework (i.e., Interpersonal Theory, Integrated Motivational-Volitional Theory, and Three-Step Theory). The 24-article Gallyer et al. review looked only at capability as conceptualised by the Interpersonal Theory. Only looking at capability from this one-theory perspective restricts identifying further evidence about capability coming to light. Whilst our review has identified that limited research has been conducted outside the Interpersonal Theory, we discuss how this is potentially hampering the theoretical progress of suicide capability.  Second, our review is systematic whereas the Gallyer et al. review is not. The Gallyer et al. appears to be a narrative review. We know this because of the method section provided by Gallyer et al., and because the authors “didn't have multiple screeners and we included extra papers as well based on what we thought would be relevant” (A.J. Gallyer, personal communication, January 14, 2022). Thus, it is possible that Gallyer et al. have actually overlooked key research that has been conducted within the Interpersonal Theory area with such an approach. A result of our broader search strategy and systematic process was the identification of a potential fourth contributor of capability, a cognitive contributor.  Third, our review looked at capability relationships with suicidal behaviours, whereas the Gallyer et al. did not, despite the authors acknowledging that these relationships are important to explore. Given the above we feel that there are significant differences between our study and the Gallyer et al. study that means that the two reviews are distinctly different. |
| First and mainly, the overall review relies on the assumption that capability for suicide can be accurately differentiated into three subfactors of acquired capability, dispositional capability, and practical capability. However, there is insufficient theoretical and empirical support for this three-factor structure as well as their validity. Aside from the validity of this approach, there is very little description provided on these three subfactors and what may be good indicators of these subfactors. Thus, the entire article appears to lack rationale for the pursuit of the exploration of the three subfactors of suicide capability, which makes me question whether the remainder of the article yields any useful information as it lacks both theoretical and empirical justification. | The purpose of a scoping review is to identify what research has been conducted in a particular broad area. For this review, the aim was to systematically capture, collate, and synthesise the empirical research that has been conducted on suicide capability within the ideation-to-action framework. Thus, we have not assumed that capability can accurately be differentiated into three sub-factors nor is the outcome of the review to “identify good indicators of these subfactors”. Rather we are seeking to simply identify and categorise what capability research has been conducted at this point in time. Given there has been theoretical development of capability within the ideation-to-action framework, no review has mapped the current state of play re research that captures this new development. Given an outcome of the review is to identify empirical support for various sub-factors, the reviewer’s point seems to be circular. If we were only to investigate factors for which there is empirical evidence, we would not be needing to do a scoping review.  Other reviews have also previously relied on the assumption that capability for suicide is only a single contributor – acquired capability. This assumption has resulted in the conclusion that capability appears more complex than a single contributor. That is, this narrow single contributor focus does not reflect the complex nature of suicide. Further, research that includes contributing factors outside of acquired capability are likely to have been overlooked in previous reviews. Therefore, to reflect the complexity of the movement from ideation-to-action we utilised Drs. May and Klonsky’s conceptualisation of capability. In doing this, it allowed us to cast a broad and wide net to identify relevant research as is consistent in conducting a scoping review. To address the reviewer’s concern, we have included the following.  “A weakness of these two theories is that they conceptualise suicide capability as consisting of individual contributors and focus on these single contributors when attempting to understand the move from ideation-to-action. Given suicide behaviour is multifaceted [14], it is important to consider contributors in combination as a single contributor is unlikely to capture the complexity that underpins the movement from ideation to attempt [15]” (lines 66-71).  “Contributors from the previous two models of suicide have been incorporated into these three overarching contributors of capability. The theory argues that an individual develops a capability for suicide through this combination of contributors [17]” (lines 74-76). |
| There is a lot of reference regarding Drs. May and Klonsky’s works on the three-step theory. However, not as much focus or consideration seems to be given to the other ideation-to-action frameworks. Most of the argument for a three-subfactor approach to the study of suicide capability appears to stem from the works of Drs. May and Klonsky. There is nothing wrong with thoroughly referencing and basing the need of a manuscript on a single other paper. However, the authors do not clearly state what about the article, what about their arguments, claims, etc., are, and how it is clearly tied to a need to pursue such a scoping review, which makes the need for this research endeavor unclear. | Thank you for this feedback. The need for this scoping review was not based on a single paper. We have now added the following to explain that the scoping review was based on “recent theoretical developments and past review limitations necessitate an up-to-date mapping of evidence relating to contributors of suicide capability and potential relationships with suicidal behaviours” (lines 119-121).  We also added limitations of previous reviews and justify further why this review is needed:  “These reviews have either been narrow in their focus (i.e., only focused on acquired capability) [18,19], narrative (i.e., potentially overlooking all and/or novel contributors) [20-22], did not explore whether suicidal behaviours were related to suicide capability [20], and/or are dated (i.e., the last systematic review was published 5 years ago) and thus may not capture more recent research. Given the timing and focus of these reviews, they do not capture studies that include other contributors of suicide capability as suggested by the Integrated Motivational and Volitional model [12,13] and the Three-Step Theory [9,16]” (lines 101-108). |
| There are also lack of clarity regarding the overall flow of logic in this manuscript as well that are confusing to follow and also make the logic behind this manuscript appear to be suboptional. For example, authors claim that the focus on a single construct (i.e., capability for suicide) is limited, then go on to explore the three domains of suicide capability. I see this as self-contradictory, as dividing up a single factor into three subfactors does not deviate from the focus on the single factor. Additionally, authors also claim the capability in all three subdomains do not need to be elevated in order for suicide to occur – I am not convinced this is accurate, as having access to lethal means do not guarantee someone will necessarily make a suicide attempt, if they are not elevated on fearlessness about death and pain tolerance. | We thank the reviewer for this comment. The reviewer may have overlooked our claim that the focus on one aspect of suicide capability, acquired capability, is limited. We agree that there is a single construct (i.e., capability for suicide), however we argue that this construct comprises more than one contributor (i.e., acquired capability). We agree with the reviewer that capability requires more than a single domain for suicide to occur. Therefore, other contributors of suicide capability need to be considered:  “One systematic review found equivocal support for acquired capability with only half of the studies providing support for its predictive ability in relation to suicide attempt [18]. In addition to this, a meta-analysis identified weak relationships between acquired capability and suicide attempts [19]. The authors suggested that acquired capability may be more complex than first proposed with other components such as genetics likely contributing to capability. Thus, suggesting that on its own acquired capability may not be sufficient to explain the movement from ideation to attempt” (lines 79-85).  “The “explosion of work in recent years” [21 p6] and recent theoretical developments of suicide capability within the Integrated Motivational and Volitional model [12,13] and the Three-Step Theory of Suicide [9,16] raise questions about the utility of previous reviews to guide future development of the construct” (lines 98-101). |
| Such lack of elaboration or logical connection is also noted in Discussion/Limitation section of the manuscript. For example, the main limitation listed by the authors (i.e., lack of empirical research that tested all three subfactors of suicide capability) is likely inevitable without the need for a systematic review – the Franklin et al. (2017) meta-analysis was not published too long ago; therefore, majority of the articles identified and included in any review will likely have the limitations of focusing on a handful of overstudied risk factors. Of course, this does not mean articles published prior to the meta analysis should be excluded – however, the interpretation of the findings need to integrate the meaning of consistent findings, changes in the literature, etc., into the discussion section. | Given we added 33 studies to the original 57 studies to now total 90 studies, the discussion has changed substantially. For example, a consistent finding is that painful and provocative events appear to be a key contributor of acquired capability, perhaps more so than fearlessness about death. This addresses the reviewer’s comment about meaning of consistent findings.  We now have two main limitations of the literature. First, the continued single factor focuses of studies “risks repeating the unproductive conclusion that almost all negative life events are risk factors for suicide [6]. This lack of differentiation amongst risk factors was a key foundation for the need for the ideation-to-action framework [5], and yet this review suggests that the existing research within the ideation-to-action framework is falling prey to the same criticism by continuing the focus on single variables.” (lines 648-652).  Our second limitation also addresses changes (or lack thereof) in the literature. “A second limitation identified is that only a small number of studies have been conducted on contributors that are not the acquired contributor. Results are promising, however the limited evidence available within each contributor raises questions about how contributors potentially function within the concept of suicide capability. Similar to previous [20,21] findings, practical contributors may play a role in suicide capability, but given the limited evidence we can only speculate about the impact that knowledge of and access to lethal means has for the movement from ideation-to-action. Adding to this uncertainty is what appears to be a sporadic and disconnected approach to building the evidence base. Study publication dates for each contributor were similar in that close to half of studies were published before 2017, raising questions about whether studies without significant results have been impacted by publication bias. Early non-significant findings that are not able to be published may lead to contributors of potential interest being abandoned in favour of known contributors that have found support and thus are more likely to be published” (lines 660-672). |
| I think it is fine to focus on empirical data that published adult samples, but I am not convinced that the authors provided a sufficient explanation. To state that emotion regulation is a “developmental factor” seems inaccurate as emotion regulation difficulties are not limited to age. “Cognitive coping strategies” may also be limited in older adults experiencing cognitive decline. | We have updated this to include children and adolescent psychosocial factors and motivations for attempting suicide:  “Children and adolescents were excluded from this review because, while children and adolescents do also attempt and die by suicide, there may be psychosocial factors that are unique to this population, such as underdeveloped emotional regulation [27] and coping skills [28] that could contribute to their capability for suicide. Further, research suggests that children and adolescent suicide attempt motivations differ significantly from adults which is often in the context of interpersonal problems [29]” (lines 138-144). |
| In sentence 49, “suicide” should be modified to “suicide death”. | Thank you. This sentence has been changed and now reads “… not every attempt will result in a death by suicide” (lines 47-48). |
| Sentence 59-61 is an incomplete sentence that needs to be revised. Same applies to sentence 347-348 and 577-578. Please thoroughly check the entire draft to make sure no other sentence structure problems exist throughout the manuscript. | Thank you. Sentence structure has been revised throughout the manuscript. |
| “US” (country name) needs to be U.S. | This has been changed throughout the manuscript. |
| The use of “thus” seems to be grammatically incorrect throughout the paper – please check grammar and sentence structure carefully when submitting a manuscript for review. | We have checked and resolved grammar and sentence structure. |
| Reviewer 2 |  |
| The authors provide a systematic review of the literature examining suicide capability in relation to suicide attempts. While I believe this is an important area for review, I found the review lacking in many ways. The biggest issue I have is the unclear definition or conceptualization of suicide capability used in the review inclusion / exclusion criteria. This makes it very hard to clearly understand what this review contributes to the literature, and, moreover, how the authors determined which studies would be included in the review, impacting my ability to determine whether or not the review presents a comprehensive picture of the capability literature.  Further, as the review currently stands, it seems there is a great level of emphasis on the details of each study (i.e., age, ethnicity of sample) and far less on the studies primary findings, limiting the impact this review can have. I provide other points of feedback below. | Thank you for bringing this to our attention. We have clarified the inclusion / exclusion criteria, so the reader understands where we “drew the line” with regard to article inclusion. For example:  “Research studies needed to include a result relating to suicide capability and suicide attempt or death by suicide. This could be results that compared groups (i.e., suicide attempters and/or deaths by suicide to controls and/or suicide ideators), or results that indicated how suicide capability was associated with suicide attempts and/or deaths by suicide through correlational or qualitative studies.” (Table 1, line 189).  As the manuscript is a scoping review, we provide an overall ‘map’ of the field. This includes both the details of each article and what each article primary findings contribute to suicide capability knowledge. That said, we changed the way we present the findings of each article (i.e., based on research design), so it is clearer than the previous submission. |
| I highly recommend updating review – currently 1 year old and there has been notable, important research since. | Thank you, we agree. The review has been updated with the amended search and was completed on 17 January 2022 (line 230). The amended search is discussed in a later comment below. |
| Introduction feels a bit long and meandering given the straightforward and salient nature of acquired capability in the suicide literature; overall is a bit wordy and repetitive. I feel it could be streamlined. | Thank you. We have streamlined the introduction. |
| The rational for the current review could be strengthened. That is, it does not seem sufficient to say a review is needed simply because there has not been one (or has not been one in the past 5 years).There are several reasons for which a review of this nature may be useful (i.e., recent research criticizing the conceptualization of capability), they should just be more clear. | Thank you. We have strengthened the rationale beyond the lack of a recent review.  For example, we discuss the Interpersonal Theory and Integrated Motivational-Volitional Theory conceptualisations of suicide capability:  “A weakness of these two theories is that they conceptualise suicide capability as consisting of individual contributors and focus on these single contributors when attempting to understand the move from ideation-to-action. Given suicide behaviour is multifaceted [14], it is important to consider contributors in combination as a single contributor is unlikely to capture the complexity that underpins the movement from ideation to attempt [15]  We also discuss the limitations of previous reviews and justify why this review is needed:  “These reviews have either been narrow in their focus (i.e., only focused on acquired capability) [18,19], narrative (i.e., potentially overlooking all and/or novel contributors) [20-22], did not explore whether suicidal behaviours were related to suicide capability [20], and/or are dated (i.e., the last systematic review was published 5 years ago) and thus may not capture more recent research. Given the timing and focus of these reviews, they do not capture studies that include other contributors of suicide capability as suggested by the Integrated Motivational and Volitional model [12,13] and the Three-Step Theory [9,16]” (lines 101-108). |
| Curious as to why the authors used “AND” with suicide* and attempt* - wouldn’t this limit research that speculates about lethal suicidal behavior, or studies that use the term suicidal behavior versus attempt? | This comment markedly helped identify additional studies and we are appreciative for the reviewer’s curiosity. We amended our search to include behaviour in addition to attempt:  suicid*  AND  attempt* OR behaviour* OR behavior*  AND  capa* OR “access to means” (line 228). |
| While necessary, all of the inclusion criteria centered around suicide attempts, rather than being related to suicide capability. Did the authors consider the definition of suicide capability in each study? Were the studies required to directly report that a measure was an indicator of capability, or were studies included that used measures hypothesized to be an indicator of the capability, even if the article itself did not discuss the measure in those terms (i.e., pain tolerance; childhood maltreatment)? Based on the inclusion criteria I don’t have a good sense of what type of studies, as it pertains to capability, were included, or where the authors “drew the line”, so to speak, with regard to study inclusion. | Thank you for bringing this to our attention. We have updated the inclusion / exclusion criteria in Table 1 (line 189):  “Direct measure of suicide capability (e.g., acquired capability for suicide scale) or measures hypothesised to contribute to suicide capability (e.g., pain tolerance)”. |
| Low quality studies were reported to use “psychometrically unsound measures”. How was this determined? I ask as it is highly relevant to the current review and the use of the Acquired Capability for Suicide Scale, which has had debated psychometric properties. | We have updated the review to make it clear how quantitative study quality was determined:  “Low quality study issues centred on measures that were psychometrically questionable (e.g., using incomplete measures or individual items from measures), and/or confounding factors (e.g., gender) not being identified or no mention of statistical strategies (e.g., matching of participants [53]) being used to deal with those factors. These same confounding factors were also common in quantitative medium quality studies, but statistical strategies were included to deal with those factors thus differentiating low and medium quality studies” (lines 246-252). |
| I would like to see more discussion of how the findings of the review fit within the broader literature – what does this means for the field going forward? How should we do better, etc.? | Thank you. We have updated the discussion and provide three recommendations for future research to improve limitations of current research. The recommendations are briefly shown below.  First, we recommend “studies that include multiple contributors are suggested to better reflect the ideation-to-action framework foundation and are more likely to assist in advancing suicide capability as a multifaceted concept” (lines 707-709).  Second, “quantitative studies should replicate and expand on the various painful and provocative events identified in this review to consolidate and generate new knowledge about suicide capability” (lines 719-721).  Finally, “future research will benefit from using psychometrically sound measures rather than partial versions to allow accurate interpretations and conclusions to be drawn across studies. Moreover, using the same scales is necessary for replication studies and continuity for theory development [159]” (lines 731-734). |
| I suggest the authors carefully read the manuscripts as there are several awkward phrases that are also inaccurate (i.e., …acquired contributor refers to…the reduction or elimination of fearlessness about death.). | Thank you. We have read and revised the manuscript. |
