# Supplementary material for: Suicide capability within the ideation-to-action framework: A systematic scoping review
Source: PLoS One. 2022 Oct 27;17(10):e0276070. doi: 10.1371/journal.pone.0276070 (PMC9612581; doi:10.1371/journal.pone.0276070)
Supplement: Attachment — Submitted filename: Response to Reviewers.docx [file pone.0276070.s004.docx]

Response to Reviewers

We thank the reviewers for their encouraging and insightful comments. Please see our responses below.

| Reviewer 1 comments | Author’s response |
| --- | --- |
| Firstly, in the introduction section authors argued for the relevancy of their scoping review, mostly comparing it to other forms of reviews. But, as these other reviews were often systematic reviews, I argue that no equivalency exists between the scoping review presented here and the systematic reviews referenced. For example to call a systematic review "to narrow" (page 5 line 101) in comparison to a scoping review is a non-equivalent comparison, as both have different aims. The paper would be stronger and more accepted by the broader scientific community, if the authors would refrain from attacking these other forms of reviews. The fact that no (recent) scoping review exists to this research question and why it should exist, would be more than enough to argue for publication.  An argument which was well executed by the authors, but is muddled by the imprecise use of the term review, if each referenced review would be specified within the paper (e.g. systematic review, practitioners review…) we would all get a clearer picture of why this scoping review is needed and how it adds to the broader research field. | We agree with the reviewer that systematic reviews and scoping reviews have different aims. To quote the Joanna Briggs Institute (<https://jbi-global-wiki.refined.site/space/MANUAL/4687794/11.1.1+Why+a+scoping+review%3F>):  “If the authors have a question addressing the feasibility, appropriateness, meaningfulness or effectiveness of a certain treatment or practice, then a systematic review is likely the most valid approach (Pearson 2004, 2005). A diverse suite of approaches to conducting systematic reviews to answer different types of clinical questions (i.e., effectiveness, prognosis, risk, etc) exist (Munn et al. 2018b). However, authors do not always wish to ask single or precise clinical questions and may be more interested in the identification of certain characteristics/concepts in sources of evidence, and in the mapping, reporting or discussion of these characteristics/concepts. In these cases, a scoping review is the better choice.”  Thus, systematic reviews ask single or precise questions and have a narrow focus as we have stated in the introduction. The example provided by the reviewer has been quoted incorrectly. Nowhere in the paper is the term “to narrow” used. The correct quote from the paper is: “these reviews have either been narrow in their focus …”. We have amended this sentence on page 5, lines 119-120 to now read: “These reviews have either been narrow in their focus as is consistent with the aims of a systematic review (i.e., only focused on acquired capability) [18,19] …”. This sentence now reflects the limitations inherent in any systematic review. We point out in the paper that this is narrow because the review only focused on a single contributor of suicide capability. Our review is broader because it includes more than a single contributing factor of suicide capability.  Whilst we appreciate the reviewer’s comment, we do not consider pointing out the limitations of other reviews, either the methodology or focus, is attacking. As with any review, it is imperative to outline limitations of previous reviews to identify research gaps. According to PLOS ONE guidelines (<https://journals.plos.org/plosone/s/submission-guidelines#loc-introduction>), the introduction should “note any relevant controversies or disagreements in the field”. Perhaps the word “attacking” was a poor choice of words from the reviewer. Especially so given the authors of the most recent preprint narrative review [20] identified that potential relationships between suicidal behaviour and suicide capability was not addressed in their review, but is an important area to focus on, which our review specifically addresses.  We argue that the fact a scoping review does not exist is insufficient justification for completing a scoping review. The limitations of the previous reviews are that they had a narrow focus (i.e., limited to the IPTS) and/or were dated. We have amended the sentence on page 5, lines 124-126 to now read: “Given the timing and focus of these reviews, they do not and/or could not capture studies that include other contributors of suicide capability as suggested by the Integrated Motivational and Volitional model [12,13] and the Three-Step Theory [9,16].” This review fills that gap. That is why this review demonstrates equivalency with other reviews.  The types of reviews referenced are specified in the paper (i.e., “systematic [18]” on page 4, line 95, “meta-analysis [19]” on page 4, line 97, or “narrative [20,21]” on page 4, line 101, and page 5, line 110, respectively). |
| Secondly, in the discussion section authors argue that the field does not reflect the multifaceted nature of the research question and that most studies used single factors. I think this conclusion is to hash.    As a major limitation of scoping reviews is the large amount of potential oversight inherent in these very broad searches. Using the FAD as an example, authors stated they found four studies (Line 396). My (systematic) overview of the FAD papers, all including suicide ideation and suicide attempts, has around thirty entries, of which maybe five to ten are of adolescent samples. This discrepancy in numbers shows the inherent limitation of scoping reviews. As scoping reviews use very general search strings they tend to over-proportionally miss multivariate studies, as the naming of multivariate studies often do not directly reference all used variables. I therefore find the conclusion of the authors a bit harsh regarding to few multivariate approaches, given that scoping reviews have an inherent tendency to overlook these kinds of studies. This weakness should either be addressed the limitation or discussion section. A potential way to limited the impact of such oversights, or strengthen it, would be to use the systematic reviews cited in the introduction section, to fill up found studies and contextualise study results. | This conclusion is a key finding of this review and reflects the research synthesised and included in this scoping review. Most studies within this review only looked at one contributing factor of suicide capability. This needs to be made explicit because it has ramifications for the field moving forward. Further, this finding is similar to the seminal Franklin et al. (2017, p.217) 50-years of suicide research meta-analysis concluded:  “Specifically, conclusions about the existing literature are constrained by at least three major methodological limits: (a) long follow-up intervals; (b) risk factors measured in isolation rather than in combination; and (c) risk factors measured in a static or trait-like fashion.”  We do not believe our conclusion is too harsh, rather it simply reflects the findings of the current scoping review.  We would like to address the FAD papers examples in two respects. First, our review identified four papers that only looked at FAD, and an additional 20 papers that used FAD and painful and provocative events within the acquired contributor. This means that 24 articles are included in this review that looked only at FAD or FAD within the acquired contributor. Thus, producing similar results to the reviewer’s overview of FAD papers that they refer too.  Second, we cannot find the reviewer’s systematic review of FAD papers that they reference. If the reviewer is referring to this paper ([https://utheses.univie.ac.at/detail/55044#](https://utheses.univie.ac.at/detail/55044)), unfortunately only the Abstract is in English so we cannot read the full paper to see if this is what the reviewer is referring to in their comments. We have identified one published review authored by the reviewer (<https://doi.org/10.1192/bjo.2022.526>). Given both of these papers appear to focus on suicidal ideation and/or suicide attempts there should be discrepancies between our findings and that of the reviewer.  Our review focused on research that included suicide attempts and deaths by suicide. As such, studies that focused only on suicidal ideation would have been excluded as per our eligibility criteria. Thus it is possible that our review identified fewer FAD papers because of our exclusion criteria that reflects the purpose of our review.  We respectfully disagree with the reviewer that very broad searches in scoping reviews equates to oversight of multivariate research. We could not find such a limitation of scoping reviews in the published literature. With that we welcome the reviewer providing us with a reference that states this and/or a reference that outlines that scoping reviews by their very nature miss multivariate studies.  Two narrative review reference lists were utilised in the search strategy for our scoping review as stated on page 8, lines 197-198. It should be noted that the systematic review [18] and the meta-analysis [19] both concluded that suicide capability as conceptualised by the IPTS is insufficient and is likely to be more complex and involve other factors. Thus, we see that our findings are commensurate with previous work that has not taken such a broad view of capability. Our broad search strategy did pick up several of papers that were included in the systematic review (e.g., Smith et al., 2010; Shelef et al., 2014) and the meta-analysis (e.g., Smith et al., 2013; Chu et al., 2018). Hence, we do not believe that there is evidence that there is an oversight in our findings. |
| Reviewer 2 comments |  |
| I suggest authors expand on the definitions/contributions of dispositional and practical factors to the suicide capability model on page 4 of the introduction, in a manner similar to how authors introduce acquired capability for suicide. | Thank you for this suggestion. We have separated the paragraph to highlight the difference of the Three-Step Theory allowing for the expansion of dispositional and practical contributors on page 4, lines 84-89:  “More recently suicide capability has been conceptualised within the Three-Step Theory of Suicide [9,16], as consisting of three contributors: acquired [10,11], dispositional, and practical. Dispositional contributors refer to genetics, temperaments, and personality factors that may increase or decrease capability [16]. For example, being born with low sensitivity to pain may increase suicide capability [9]. Practical contributors are those that increase knowledge of and access to lethal means [21]. An example is an individual who is well acquainted with firearms because they have both the experience with and access to a lethal means, thus increasing their capability for suicide.” |
| Given the lack of diversity (racial/ethnic, gender, SES) in majority of the articles discussed in the review, this should be added as a limitation. Further, exploring these constructs among more diverse populations that consider events that are shown to be painful and provocative for minority populations (e.g., discrimination) needs to be considered as an area for future research. | Thank you for bringing this to our attention. We agree and have added this in the limitation and future research sections.  Limitation of the literature has been added on page 54, line 818-819: “Third, most studies lacked diversity (e.g., racial/ethnicity) as the majority were U.S. participants that identified as Caucasian”.  Future research addition on page 56, lines 858-863: “Given the lack of diversity in most studies, it is suggested that exploring suicide capability among more diverse populations is warranted. There may be painful and provocative events specific to minority populations (e.g., discrimination) that contribute to capability which are not yet considered within theoretical models. Further, additional research across the world is required to develop understanding about potential geographic contributors beyond firearms in the U.S.” |
