# Supplementary material for: Suicide capability within the ideation-to-action framework: A systematic scoping review
Source: PLoS One. 2022 Oct 27;17(10):e0276070. doi: 10.1371/journal.pone.0276070 (PMC9612581; doi:10.1371/journal.pone.0276070)
Supplement: Attachment — Submitted filename: Response to Reviewers.docx [file pone.0276070.s005.docx]

Response to Reviewers

We thank the reviewers for their helpful comments. Please see our responses below.

| Reviewer 3 comments | Author’s response – *N.B.* page and line numbers refer to the manuscript with track changes accepted. |
| --- | --- |
| The topic is interesting but the ms needs revisions before being considered for publication. For example formatting and position of the tables should be reconsidered. As it is now the ms is very long and sometimes difficult to follow. | We appreciate this this overall comment and have repositioned Tables 3 and 4. |
| page 101 line 82 and followings. Please give some information on the acquired factors. | Yes, thank you. We have added painful and provocative event examples as we have included examples for the dispositional and practical contributors.  Please see page 3, line 62 for the change.  (e.g., childhood maltreatment, combat exposure [11]) |
| "The templated" check whether correct | Thank you for pointing this out. We have amended this to “template” as seen on page 9, line 200. |
| "(see Fig 1)" I think that "Figure" should be included and not "Fig" | The journal guidelines state, “Cite figures as “Fig 1”, “Fig 2”, etc.” Therefore, Fig 1 has not been changed. |
| Page 124 Please check the sentence "Most studies focused on individuals without a psychiatric" | Thank you for this raising this with us. Because Tables 3 and 4 intersect the text at this point, the sentence appears incomplete when it is not an incomplete sentence. We have moved Tables 3 and 4 to be positioned after the paragraph this sentence is in. This increases readability which the Reviewer commented on, whilst adhering to the journal’s formatting guidelines.  Please see location of the tables which now start on page 26. |
| page 145 Please check the sentence "was found to be related with suicide attempt history [118] more so than individuals who had" | Thank you for this comment. We have amended the sentence on page 45, lines 462-464, to now read:  “Yet NSSI frequency (i.e., frequency, severity, and duration of NSSI) was found to be related to individuals with suicide attempt history [118] more so than individuals who had not attempted suicide [119].” |
| Please check the sentence "Three association studies found cognitive difficulties were reported" | We have changed the word “difficulties” to “deficits” to reflect an earlier sentence in this paragraph. Please see page 48, line 544 for this change. |
| Please check "ambiguity about how these two constructs potentially effect acquired capability." | Thank you for bringing this to our attention. We have changed the sentence on page 52, lines 649-651, to now read:  “This lack of combination may be adding to the ambiguity about how these two constructs potentially contribute to the development of acquired capability”. |
